# T cells in idiopathic pulmonary fibrosis: crucial but controversial

**DOI:** 10.1038/s41420-023-01344-x

**Published:** 2023-02-14

**Authors:** Lishan Deng, Teng Huang, Lei Zhang

**Affiliations:** Department of Respiratory and Critical Care Medicine, NHC Key Laboratory of Respiratory Diseases, Tongji Hospital, Tongji Medical College, Huazhong University of Science and Technology, 1095 Jiefang Ave, Wuhan, 430030 China

**Keywords:** Cystic fibrosis, T-cell receptor

## Abstract

Idiopathic pulmonary fibrosis (IPF) has been extensively studied in recent decades due to its rising incidence and high mortality. Despite an abundance of research, the mechanisms, immune-associated mechanisms, of IPF are poorly understood. While defining immunopathogenic mechanisms as the primary pathogenesis is controversial, recent studies have verified the contribution of the immune system to the fibrotic progression of IPF. Extensive evidence has shown the potential role of T cells in fibrotic progression. In this review, we emphasize the features of T cells in IPF and highlight the controversial roles of different subtypes of T cells or even two distinct effects of one type of T-cell in diverse settings, and multiple chemokines and cell products are discussed. Furthermore, we discuss the potential development of treatments targeting the immune molecules of T cells and the feasibility of immune therapies for IPF in clinical practice.

## Facts

- IPF is a heterogeneous disease characterized by an aberrantly activated immune system.
- T cells play a crucial role in IPF pathogenesis.
- Biologics targeting T-cell-related mediators (cytokines and chemokines) are potential IPF therapies.

## Open questions

- Do other subtypes of T cells play a role in IPF?
- Are there any other costimulatory molecules except CTLA4 and CD28 associated with IPF?
- What’s the mechanism of Th17 cell susceptibility to Tregs?
- What is the mechanism by which Tregs shift their roles in distinct stages of IPF?

## Introduction

Idiopathic pulmonary fibrosis (IPF) is an interstitial lung disease characterized by chronic, progressive fibrosis that is associated with a severe inexorable decline in lung function and significant mortality [1]. There is evidence suggesting that the incidence of IPF is rising [2, 3]. The morbidity of IPF is estimated to be up to 2.8–9.3 per 100,000 per year in North America and Europe, with relatively lower rates in Asia and South America [4]. Although several treatments are available to patients with IPF, the median survival of patients diagnosed with IPF is reported to be less than 3 years [3]. Despite extensive studies, the unclear etiology and mechanisms make the diagnosis and treatment of IPF complicated.

Several epidemiological studies have demonstrated diverse risk factors associated with IPF, including cigarette smoking, metal dust [5], agriculture, farming, livestock, wood dust, stone, sand, silica [6], and microbes [7]. However, individual factors at the genetic and epigenetic levels are the most important factors in the fibrotic progression of IPF [8], and their interactions with putative external factors have not been explicitly clarified. With regard to pathogenesis, repeated microdamage to alveolar epithelial tissues has been deemed the first trigger of an aberrant wound repair process, which contributes to the occurrence of fibrosis [9]. Thus, repeated repair of the dysfunctional epithelium is the key to understanding how fibrosis develops [10]. Specifically, when type 1 alveolar epithelial cells (AEC1s) encounter repeated microinjuries, type 2 alveolar epithelial cells (AEC2s) regenerate damaged epithelial cells, but due to their inability to restore them, the wound healing process promotes fibrosis with changes in the expression of cytokines, ultimately contributing to an imbalance in profibrotic and antifibrotic effects [11, 12]. For instance, TGF-β1 is considered a potent profibrotic mediator because it promotes epithelial-mesenchymal transition (EMT), extracellular matrix production, epithelial cell apoptosis and migration, the recruitment of fibrocytes and immune cells, fibroblast activation, myofibroblast proliferation, growth factor production and proangiogenic mediator production [9, 13]. TGF-β1 can promote the expression of downstream target genes, including procollagen I and III, via transmembrane receptor serine/threonine kinases and the cytoplasmic SMAD-2/3 signaling pathway [14]. A decrease in SMAD-3 has also been shown to attenuate fibrosis in IPF [15]. Furthermore, other signaling pathways, including the extracellular signal-regulated kinase (ERK), mitogen-activated protein kinase, the phosphatidyl inositol 3-kinase/Akt pathway, and Rho-like GTPase, have also been shown to play a role in TGF-β1-facilitated pulmonary fibrosis [16–18]. In addition, TGF-β1 increases the expression of vascular cell adhesion molecule 1, which is a mediator of fibroblast proliferation [19]. Progressive fibrosis induced by elevated mechanical tension is also related to the upregulation of TGF-β expression [20]. Therefore, it is clear that immune pathways may play an important role in fibrosis. Although the specific mechanisms of inflammation and immunity in IPF are still unclear, there is evidence that the immune system is aberrantly activated in IPF [21].

T lymphocytes play a key role in adaptive immunity. Mature T cells not only mediate the cellular immune response but are also associated with the thymus-dependent antigen (TD-Ag)-induced humoral immune response. T cells are derived from lymphoid progenitor cells in the bone marrow and become activated in the thymus; then, they migrate to organs through blood circulation. Depending on the presence of cell surface proteins, there are two subtypes of T cells: CD4^+^ and CD8^+^ cells, which recognize MHCII and MHCI, respectively. In the lung, CD4^+^ and CD8^+^ T cells serve as the dominant adaptive immunocytes, participating in the clearance of pathogens and then persisting as memory T cells. Intriguingly, memory T cells can persist as tissue-resident memory T cells (T_RM_ cells) [22]. Furthermore, apart from infectious diseases with definite aetiologies, many pulmonary diseases, such as asthma [23] and acute respiratory distress syndrome [24], are related to immune responses mediated by T lymphocytes to varying extents, and pulmonary fibrosis is no exception [25]. In particular, some T-cell–related genes and proteins, such as CD28 and LCK, have already been used as prognostic biomarkers in IPF [26, 27]. Specifically, transcriptional profiling of peripheral blood mononuclear cells (PBMCs) in IPF clarified the correlation between the downregulation of the T-cell regulatory gene CTLA4 and a reduction in event-free survival [28]. Analogously, there have been findings showing that decreased expression of the costimulatory molecule CD28 on circulating T cells can predict a poor outcome for IPF patients [29]. Intriguingly, a significant increase in CD8^+^ CD28^null^ T cells was discovered in IPF lung tissues, and CD28^null^ T cells express CTLA4, similar to CD28^+^ T cells. In addition, anti-CTLA4 treatment could accelerate fibrosis in IPF. Therefore, CD28^null^ T cells may promote fibrosis, but the immune checkpoint CTLA4 may be protective and limit this effect [30]. However, how T lymphocytes are associated with IPF remains unclear. In this review, we will describe the role of T cells in the progression of IPF.

## Characteristics of T cells in IPF

T cells represent a small population in the lungs in normal individuals; however, consistent with a pulmonary inflammatory response, enriched T cells are diffusely present in bronchoalveolar lavage and lung tissues in IPF [25, 31, 32]. CD4^+^ and CD8^+^ T cells play roles in IPF. Papiris et al. showed a significant increase in CD8^+^ T cells in the lung tissues and bronchial lavage fluid of patients with IPF. Furthermore, CD8^+^ T cells are related to the grade of dyspnea and functional parameters of disease severity [33, 34]. There is evidence that CD8^+^ T cells accelerate lung damage when they are recruited and react to viral infection [35]. Furthermore, CD8^+^ T cells infiltrate the parenchyma diffusely in the fibrotic tissues of IPF and can differentiate into cells that produce IFN-γ but not IL-4, leading to the attenuation of fibrosis, and cells that produce IL-4 but not IFN-γ, promoting fibrosis [36]. In brief, CD8^+^ T cells may have opposing impacts on fibrosis, but higher levels of CD8^+^ T tend to indicate more severe lung injury. With respect to CD4^+^ T cells, the expression of the chemokine receptors CXCR3 and CCR4, which are associated with Th1 and Th2 cells, respectively, indicates the possible dominance of Th2 cells in IPF [37]. Moreover, the expression of CXCR3 and CCR4 in CD4^+^ T cells in the BAL fluid of IPF patients is strikingly lopsided [38]. Therefore, a functional imbalance of the Th1/Th2 immune response was thought to play a crucial role in IPF pathogenesis. The Th1/Th2 ratio can predict the severity and prognosis of the disease to some extent [39].

IPF is a disease that affects elderly individuals, and the immune system becomes inefficient with age. The thymus is responsible for producing naïve T cells and is gradually replaced by fatty tissues in individuals over the age of sixty [40], and the adaptive immune system decays accordingly. Naïve CD4^+^ and CD8^+^ T cells are reduced in ageing lungs relative to memory T cells [41]. Compared to young normal volunteers, the CD4-to-CD8 ratio in bronchoalveolar fluid lavage is strikingly higher in elderly adults, suggesting that fewer naïve T cells can be converted to functional memory T cells with age, which is also related to a decrease in the number of DCs available for priming CD4^+^ T helper cells [42, 43]. Additionally, regulatory T cells are increased in older humans [44], but the mechanism of this change remains unclear, particularly the association with the decreased number of regulatory CD4^+^ T cells [43]. In addition, there are many other factors related to IPF pathology that affect the regulation of T cells, which requires more studies.

Indeed, an increasing number of studies in recent years have found that each specific subtype of T-cell may promote or reverse the progression of specific mechanisms. In the following text, we want to discuss the features and impact of several subpopulations of T cells, mainly CD4^+^, including Th1 cells, Th2 cells, Th17 cells, and regulatory T cells (Tregs) in IPF.

## Th1 cells

Although inflammation is considered indispensable in the progression of fibrosis, pro-inflammatory factors are not always profibrotic. The pro-inflammatory cytokine IFN-γ is typically antifibrotic because it suppresses collagen deposition by fibroblasts. Thus, Th1 cells, which are derived from Th0 cells that are activated by IL-12, are widely recognized as antifibrotic T cells due to their production of IFN-γ [45, 46]. A decrease in the levels of IFN-γ in the BAL fluid or circulation of IPF patients suggests the positive effect of Th1 cells [47]. Xu et al. further demonstrated that deficiency of the Th1 transcription factor T-bet in CD4^+^ T cells increases the susceptibility to BLM-induced lung fibrosis in BALB/c mice, which are naturally resistant to BLM [48]. Furthermore, Kass et al. [49] suggested that the increased gene expression of cytokine-like factor 1 was associated with an increase in CD4^+^Tbet^+^ T cells. Th1-type chemokine patterns in IPF showed that lung fibroblasts tend to induce a Th1-type immune response under normal conditions; that is, Th1 cells participate in the normal scar healing process [50].

## Th2 cells

In contrast to Th1 cells, Th2 cells are recognized as profibrotic in the pathology of IPF and are characterized by the production of IL-4, IL-5, and IL-13. As noted previously, Th2 cells were dominant in IPF and antagonized Th1 cells. Furthermore, a striking increase in Th2 cytokines was detected in the BAL fluid of IPF patients [51, 52]. Studies on IL-4 and IL-13 clarified that these chemokines promote the proliferation of fibroblasts and induce fibroblast differentiation into myofibroblasts, which could be inhibited by the Th1 cytokine IFN-γ [53]. In addition, Wynes et al. [54] demonstrated that IL-4 could stimulate macrophages to produce the survival factor IGF-I to protect myofibroblasts from apoptosis, and then fibrosis tends to be progressive. Given the antagonistic relationship between Th1 and Th2 cells, some researchers have attempted to treat patients with IPF with interferon gamma-1b in a placebo-controlled trial, but the survival of patients showed no improvements [55, 56]. A similar trial of tralokinumab, a human IL-13 monoclonal antibody, likewise showed no efficacy [57]. Undoubtedly, Th1 and Th2 cells play important roles in the pathogenesis of IPF and have attracted much attention from researchers; however, the notion of the Th1/Th2 imbalance as the crucial mechanism in IPF pathogenesis and strategies targeting pathogenic products may need more evaluation.

## Th17 cells

Th17 cells are characterized by the production of IL-17 and are the third subgroup of Th cells. The role of Th17 cells in IPF is not completely understood. The classical cytokines secreted by Th17 cells, IL-17 and IL-22, contribute to host defense in many infective circumstances but also accelerate the inflammatory process in multiple autoimmune diseases, such as rheumatoid arthritis [58, 59]. IL-22 shows no significant variations in patients with IPF compared to normal individuals [60]. IL-17 is not an exclusive product of Th17 cells but is also derived from a variety of other immune cells, including macrophages, neutrophils, NK cells, ILC3s, and γδ-T cells, the latter of which is particularly interesting [61, 62]. IL-17-producing cells have been identified as critical cells in mucosal immunity, including the respiratory tract [63].

Importantly, Th17 cells and IL-17 are detected in the inflammatory and fibrotic tissues of patients with IPF [64], and an increase in IL-17 is believed to have potential in the development of fibrosis. In a bleomycin-induced lung fibrosis murine model of IPF, Wilson and colleagues [65] demonstrated that IL-17 not only stimulates collagen deposition and ECM production but also mediates TGF-β signaling. In addition, they observed that IL-17A was associated with neutrophil recruitment to the lung, which is a typical change in BLM-induced fibrosis. In their study, however, the regulation of TGF-β and IL-17A was not well defined. Interestingly, Celada et al. found that PD-1^+^ Th17 cells are a significant source of TGF-β, and the upregulation of PD-1 on CD4^+^ T cells promotes the production of IL-17A and TGF-β, ultimately exacerbating fibrosis [66]. These studies associated IL-17A with PD-1 and STAT-3, but some other contributors to fibrosis may have been ignored. Furthermore, IL-17 promotes the expression of IL-6 by fibroblasts [67] and IL-8 by epithelial cells [68], resulting in neutrophilic influx. More interestingly, IL-17A antibody therapy could ameliorate fibrosis in murine models of IPF [67, 69].

Moreover, the Th17 lineage is characterized by low susceptibility to regulatory T cells (Tregs) [70]. Given that Tregs are responsible for regulating the immune response and maintaining immune tolerance, this feature is crucial for the persistence of inflammation caused by Th17 cells to a certain extent and supports the role of Th17 cells in the autoimmune process. Accordingly, the neutralization of IL-17A induces the accumulation of Tregs and alleviates silica-induced pulmonary fibrosis in murine models [71]. In mice, IL-27 administration attenuates pulmonary fibrosis by redressing the imbalance in Th17 cells and Tregs [72]. However, there is a lack of clinical trial evidence, and more studies on Th17 cells and related cytokines are needed. The stimulator of Th17 differentiation also requires more exploration.

## Regulatory T cells

Tregs play a pivotal role in maintaining immune tolerance and immune homeostasis. The role of Tregs in IPF has been extensively investigated in recent years, but the exact promotion or suppression is unclear. Studies identifying Tregs as antifibrotic have been widely reported. Among IPF patients, significant impairment of Treg function is observed in the peripheral blood and BAF, which is also related to insufficiencies in FVC and diffusion capacity [73]. In support of this view, upregulating the expression of Tregs by reducing CCR7 expression attenuates fibrosis in mice [74]. However, due to the effect of TGF-β production, which results in fibroblast proliferation, Tregs are recognized as promoting fibrosis progression [75, 76]. A significant increase in the proportion and absolute quantity of Tregs in IPF patients has been reported in recent studies, and these patients are characterized by dysregulation of the Treg/Th17 axis [77]. An imbalance in Treg subtypes, especially an increase in the proportion of activated Tregs, is also correlated with the severity of IPF [78]. Sema 7a^+^CD4^+^CD25^+^FoxP3^+^ Tregs are one of the typical aberrant subtypes and can induce TGF-β1-mediated fibrosis as a result of suppression impairment [79]. In addition, reversing Treg differentiation by inhibiting the PD-1 pathway ameliorates fibrosis by reducing collagen-1 accumulation [80].

Intriguingly, Daniel et al. [81] demonstrated that the contradictory roles of Tregs depend on the stage of pulmonary fibrosis. In a bleomycin-induced lung fibrosis model, Tregs may mediate TGF-β1 generation and collagen accumulation during the injury phase but might ameliorate pathology in the later stages [81]. In brief, Tregs play contradictory roles in the progression of IPF, and the exact promotion or inhibition depends on the disease stage. Further experiments to clarify whether Tregs attenuate fibrosis in vivo are needed, as murine models do not completely recapitulate human IPF.

## Summary and perspective

**Fig. 1:**
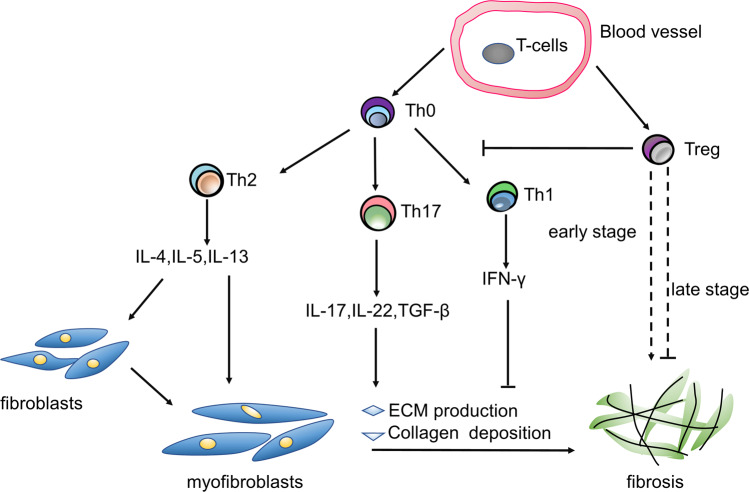
Graphic illustration of important T-cell subtypes in IPF. When the immune system is stimulated by external risk factors, T cells migrate to the interstitium and play different roles as a result of various secreted chemokines. Th1 cells are antifibrotic due to the suppressor IFN-γ. Th2 cells can activate fibroblasts and myofibroblasts. IL-17 produced by Th17 cells extensively promotes ECM production and collagen deposition. Tregs inhibit immunoreaction and may have opposing functions in the early and late stages of fibrosis. Th T helper cell, Treg regulatory T-cell, TGF transforming growth factor, IFN interferon, ECM extracellular matrix.

In summary, compared to those in healthy subjects, the quantity or dominant chemokines produced by each subtype of T cells in IPF patients are changed (Table 1). Although the exact pathogenesis of IPF remains unclear, T cells contribute to the progression of fibrosis, and undoubtedly, the mechanisms are complicated (Fig. 1). Generally, the profibrotic or antifibrotic role of T lymphocytes in IPF varies with the subpopulations of T cells and stages of pathology. Even a traditional protective cell type may be detrimental in some circumstances. It is important to realize that T-cell-induced inflammation is essential for injury recovery, and an imbalance between inflammation and inflammation resolution can result in fibrosis [62].

**Table 1:** Comparing chemokines produced by T cells in IPF patients to those in healthy subjects.

Subtype	Normal subjects	IPF patients
Th1	TNF-α, IFN-γ, IL-2,	IFN-γ ↓
Th2	IL-4, IL-5, IL-6, IL-10	IL-4 ↑ , IL-5 ↑ , IL-13↑
Th17	IL-17, IL-21, IL-22	IL-17 ↑ , TGF-β ↑ , IL-22
Treg	IL-10, TGF-β	TGF-β ↑

Unlike directly observing fibroblast proliferation or epithelial cell injury, T-cell studies tend to be more nuanced and vary among individual immune microenvironments. Crucial molecules make it possible to diagnose IPF in the early stage, and molecular therapies targeting specific T cells or their products to restore the balance among each subpopulation of T cells can provide new methods for IPF treatment. A universal approach to T-cell-associated therapies does not apply. Therefore, precision medicine using genomics, biomarkers, and immune molecule profiles is urgently needed for the diagnosis and treatment of IPF patients.

## Data Availability

Correspondence and requests for data should be addressed to Lei Zhang.
