# Correction to: T cells in idiopathic pulmonary fibrosis: crucial but controversial

**DOI:** 10.1038/s41420-023-01375-4

**Published:** 2023-02-23

**Authors:** Lishan Deng, Teng Huang, Lei Zhang

**Affiliations:** Department of Respiratory and Critical Care Medicine, NHC Key Laboratory of Respiratory Diseases, Tongji Hospital, Tongji Medical College, Huazhong University of Science and Technology, 1095 Jiefang Ave, Wuhan, 430030 China

**Keywords:** Cystic fibrosis, T-cell receptor

Correction to: *Cell Death Discovery* (2023) 9:62 10.1038/s41420-023-01344-x, published online 14 February 2023

The original version of this article contained errors in the references. During the proof-reviewing process, new references were inserted to the manuscript. However, the authors did not know how to modify the order of references, resulting in problems in the order of references in the final version. The original article has been corrected.

